# PD-1/PD-L1免疫检查点抑制剂在肺癌中的研究进展

**DOI:** 10.3779/j.issn.1009-3419.2019.06.07

**Published:** 2019-06-20

**Authors:** 娣 张, 架旗 黄, 初峰 张, 燕 管, 其森 郭

**Affiliations:** 1 250100 济南，山东大学附属山东省肿瘤医院，山东大学 Department of Oncology, Shandong Cancer Hospital Affiliated to Shandong University, Shandong University, Jinan 250100, China; 2 250117 济南，山东大学附属山东省肿瘤医院，山东省医学科学院，山东省肿瘤防治研究院 Department of Oncology, Shandong Cancer Hospital Affiliated to Shandong University, Shandong Academy of Medical Sciences, Shandong Cancer Hospital and Institute, Jinan 250117, China

**Keywords:** 程序性死亡受体1, 程序性死亡配体1, 免疫检查点抑制剂, 免疫治疗, 肺肿瘤, Programmed death 1, Programmed death-ligand 1, Immune checkpoint inhibitors, Immunotherapy, Lung neoplasms

## Abstract

近年来，免疫治疗的研究成果突出，多项临床试验捷报频传。目前，肺癌的免疫治疗研究已取得重大突破，特别是程序性死亡受体1（programmed death 1, PD-1）或程序性死亡配体1（programmed death-ligand 1, PD-L1）免疫检查点抑制剂（如Nivolumab、Pembrolizumab、Atezolizumab、Durvalumab和Avelumab等）在各项临床试验的成功应用，为包括鳞癌、腺癌和小细胞肺癌在内的多种病理类型肺癌患者均带来了临床获益。本文通过回顾并解读具有代表性的重要临床研究，全面分析PD-1/PD-L1免疫检查点抑制剂在肺癌中的应用价值和地位。基于各项大型临床试验的探索成果，肺癌免疫治疗细节正在不断优化，肺癌免疫治疗适应证也在不断拓宽。然而，免疫治疗仍面临很多挑战，例如免疫联合策略的选择、生物标志物的探索、不良反应的管理、驱动基因突变人群的应用可行性等等。本文就肺癌免疫治疗的最新重要进展作一系统综述，以期为广大临床工作者提供前沿参考。

## Abstract (en)

In recent years, research on immunotherapy has made great progress. Currently, immunotherapy has made significant breakthrough, especially programmed death 1/programmed death-ligand 1 (PD-1/PD-L1) checkpoint inhibitors (e.g, Nivolumab, Pembrolizumab, Atezolizumab, Durvalumab and Avelumab, etc.) have brought clinical benefits to patients with various pathological types of lung cancer, including squamous cell carcinoma, adenocarcinoma and small cell lung cancer. In this paper, the application value and current status of PD-1/PD-L1 checkpoint inhibitors in lung cancer were comprehensively analyzed by reviewing and interpreting representative clinical studies. Based on the results of various large-scale clinical trials results, the indications of immunotherapy in lung cancer have been continuously broadened, and the details of immunotherapy have also been constantly optimized. However, immunotherapy still faces many challenges, such as the selection of immune combination strategies, the exploration of biomarkers, the management of adverse events, the feasibility of application of driver gene mutation population and so on. In this article, we made a systematic review about the latest progress of PD-1/PD-L1 checkpoint inhibitors in lung cancer, in order to provide cutting-edge reference for the clinical workers.

肺癌是最常见的恶性肿瘤，也是癌症死亡的主要原因。据最新统计数据表明，肺癌发病率占癌症总发病人数的11.6%，死亡率占癌症总死亡人数的18.4%，严重威胁着人民的健康^[1]^。近年来，随着检测技术和治疗水平不断提高，晚期肺癌的治疗取得重要突破，已由传统化疗时代发展到精准分子靶向治疗时代，继而发展到免疫治疗时代。目前，免疫治疗成为研究的热点，已经成为晚期肺癌治疗不可或缺的一部分，特别是PD-1/PD-L1免疫检查点抑制剂的应用取得令人鼓舞的成果。本文就PD-1/PD-L1抑制剂在晚期肺癌治疗中的研究进展作一系统性综述。

## 1 肿瘤免疫治疗

免疫系统能够识别和清除新出现的“非已”成分，包括肿瘤细胞。发生基因突变的肿瘤细胞能否形成肿瘤取决于肿瘤细胞与免疫系统之间相互作用的结果^[2]^。肿瘤的免疫治疗通过激发和增强机体的免疫功能或调节其免疫状态以达到控制和杀灭肿瘤细胞的目的，其理论基础是免疫监视和免疫编辑。Chen等^[3]^认为从肿瘤细胞释放抗原开始至免疫系统杀死肿瘤细胞结束是一个循环过程，将其定义为癌症-免疫周期，并分为七个步骤：肿瘤细胞表达和释放肿瘤抗原；处理和呈递肿瘤抗原；启动和激活T细胞；T细胞迁移至肿瘤部位；T细胞渗透至肿瘤部位；T细胞识别肿瘤细胞；T细胞杀死肿瘤细胞。这些在癌症-免疫周期中发挥作用的众多因素也为免疫治疗提供了广泛的潜在治疗靶点，其中作用于PD-1/PD-L1的免疫检查点抑制剂主要通过促进最后一个环节发挥抗肿瘤作用。

## 2 PD-1/PD-L1免疫检查点抑制剂

PD-1是CD28超家族成员的免疫抑制分子。PD-1主要表达于激活的CD4^+^T细胞、CD8^+^T细胞、自然杀伤T细胞、B细胞和活化的单核细胞，由T细胞受体或B细胞受体信号通路诱导，并在肿瘤坏死因子刺激下增强^[4]^。其主要配体为PD-L1、PD-L2。其中，PD-L1广泛表达于T细胞、B细胞、树突状细胞、巨噬细胞等多个组织中，可在促炎性细胞因子激活下进一步上调^[5]^。在机体正常情况下，PD-1/PD-L1通路发挥着重要的免疫调节作用。当肿瘤细胞上高表达的PD-L1与T细胞上的受体PD-1结合时，可传递负性调控信号，诱导T细胞凋亡或导致免疫无能，使肿瘤细胞得以逃避机体的免疫监控和杀伤。同时，PD-1/PD-L1通路激活还可改变T细胞分化，使效应T细胞（effector T cell, Teff）及记忆T细胞（memory T cells, Tm）分化受损，调节性T淋巴细胞（Regulatory T cells, Treg）和衰竭T细胞（exhaustion T cells, Tex）分化上调，从而显著地抑制T细胞免疫效应。当PD-1与PD-L1结合被阻断时，负性调控信号亦被阻断，T细胞可恢复其活性，重新获得杀伤肿瘤细胞的能力^[6]^。

PD-1/PD-L1免疫检查点抑制剂不同于传统肿瘤治疗手段，而是利用机体自身的免疫系统发挥杀伤肿瘤的作用。目前已在多种实体肿瘤和血液系统恶性疾病中彰显出卓越的疗效，其最大优势是产生持久应答、带来长期生存^[7-11]^。在肺癌领域中，针对PD1/PD-L1靶点的药物主要有5种，包括抗PD-1单抗（Nivolumab、Pembrolizumab）、抗PD-L1单抗（Atezolizumab、Durvalumab、Avelumab）。随着对肿瘤免疫机制的认识不断深入，PD-1/PD-L1抑制剂在挑战与机遇中砥砺前行，多种免疫药物的问世及大型临床研究数据的更新为肺癌的精准化治疗带来更多的选择和希望。

## 3 PD-1/PD-L1免疫检查点抑制剂在晚期NSCLC中的应用

非小细胞肺癌（non-small cell lung cancer, NSCLC）约占肺癌的80%-85%，大约有30%-40%患者就诊时已进入Ⅲ期/Ⅳ期而失去手术机会。因此，在现有的治疗方案基础上进一步提高疗效和延长生存期一直是肺癌治疗前进的方向。

### 3.1 新辅助治疗

超过三分之一的NSCLC患者在诊断时处于Ⅲ期，对于这类患者需采用多模式治疗方案。单独手术切除或放疗预后差；当累及N2淋巴结，常伴有微转移病灶，与单纯的新辅助化疗相比，新辅助放化疗不能提高生存率^[12]^。在过去的20年里，Ⅲa期NSCLC的治疗一直处于平台期。

Ⅱ期NADIM研究尝试用化疗联合免疫新辅助治疗Ⅲa期可切除NSCLC。研究选择组织学或细胞学证实Ⅲa期（N2、T4N0/N1）表皮生长因子受体/酪氨酸激酶受体（epidermal growth factor receptor/anaplastic lymphoma kinase, EGFR/ALK）阴性的NSCLC患者，行3周期新辅助治疗（Nivolumab联合紫杉醇+卡铂），第3周或4周进行手术，于3周-8周后再进行辅助治疗（Nivolumab），随访得到较好的临床缓解率（完全缓解为10%，部分缓解为60%，稳定为30%，未观察到疾病进展），并观察到了前所未有的高病理缓解率（主要病理缓解为95%)，这使新辅助化疗联合免疫成为Ⅲa期NSCLC具有前景的治疗方法^[13-16]^。但是也存在一定的局限性，目前的结果仅限于对次要终点如客观缓解率（objective response rate, ORR）和病理缓解率的初步分析，病理缓解能否转化为生存获益还有待于大样本的临床研究证实。

### 3.2 一线治疗

根据美国国立综合癌症网络（National Comprehensive Cancer Network, NCCN）指南，驱动基因突变阳性的晚期NSCLC，首选靶向治疗；而驱动基因突变阴性的患者，选用含铂类的双药治疗方案±维持治疗。尽管化疗可延长患者的生存时间，但是单纯化疗疗效达到瓶颈，ORR约15%-32%，中位总生存期（median overall survival, mOS）8.1个月-10.3个月。许多临床试验相继展开，旨在评估PD-1/PD-L1抑制剂在晚期NSCLC一线治疗的临床价值。

#### 3.2.1 一线免疫单药治疗

CheckMate026研究旨在对比Nivolumab与含铂化疗一线治疗PD-L1阳性（TPS≥1%）Ⅳ期或复发性NSCLC的疗效。结果显示，Nivolumab在PD-L1阳性（TPS≥5%）的患者中未能延长无进展生存期（progression-free survival, PFS）和总生存期（overall survival, OS）（PFS：4.2个月*vs* 5.9个月，HR=1.15，95%CI: 0.91-1.45，*P*=0.25；OS：14.4个月*vs* 13.2个月，HR=1.02，95%CI: 0.80-1.30）^[17, 18]^。但是，探索性分析发现Nivolumab在高肿瘤突变负荷（tumor mutation burden, TMB）患者中改善了ORR（47% *vs* 28%）和PFS（9.7个月*vs* 5.8个月），这是首次在Ⅲ期临床研究中证实TMB对于免疫治疗的预测价值^[19]^。

不同于Nivolumab，Pembrolizumab在晚期NSCLC一线治疗中展示出临床获益。Ib期KEYNOTE001研究发现，Pembrolizumab在初治和经治NSCLC患者中均观察到抗肿瘤活性，安全性可控。对于PD-L1阳性（TPS≥50%）初治的NSCLC，ORR和24个月OS分别达到58%和61%^[20, 21]^。基于以上结果，KEYNOTE024研究将其用于一线治疗PD-L1（TPS≥50%）、EGFR/ALK阴性的晚期NSCLC。结果证实，对比含铂化疗方案，Pembrolizumab将死亡风险降低了40%，明显改善PFS（10.3个月*vs* 6.0个月）和ORR（45% *vs* 28%）。尽管化疗组患者超过50%以上交叉接受Pembrolizumab治疗，Pembrolizumab组的OS仍显著改善（HR=0.60），且治疗相关的不良事件（advent events, AEs)发生率明显降低^[22-24]^。这使得Pembrolizumab于2016年获批成为一线治疗PD-L1高表达（TPS≥50%）、EGFR/ALK阴性的转移性NSCLC的新标准。对比发现，尽管试验设计类似，KEYNOTE024研究和CheckMate026研究存在许多不同：在活检组织上，KEYNOTE024选用未经放疗的新鲜组织，CheckMate026选用石蜡包埋肿瘤组织或随机前6个月以内未经过系统性治疗的存档标本；在PD-L1检测抗体及截断值上，KEYNOTE024选用Dak022C3、PD-L1强阳性（TPS≥50%），CheckMate026选用28-8、PD-L1阳性（TPS≥1%）。这些都可能是导致两项研究结果截然不同的重要原因。在KEYNOTE024基础上，KEYNOTE042研究在PD-L1（TPS≥1%）人群中进一步探索。结果显示，Pembrolizumab较化疗显著提高OS，PD-L1高表达者（TPS≥50%）疗效更突出；尽管暴露时间更长，Pembrolizumab治疗相关的AEs发生率更低，其安全谱与既往研究一致^[23, 25]^。

BIRCH研究在一线治疗晚期NSCLC的数据结果也显示出Atezolizumab具有良好的疗效及安全性^[26]^。此外，Avelumab单药用于一线治疗PD-L1阳性晚期NSCLC的JAVELIN Lung 100试验正在进行中。

#### 3.2.2 一线免疫联合化疗

多项全球大型临床试验相继展开免疫联合方案治疗晚期NSCLC有效性及安全性的探索，以期进一步扩大受益人群。主要联合治疗策略包括免疫联合化疗、免疫双药联合、免疫联合靶向治疗、免疫联合放疗等。其中，免疫联合化疗已取得重大突破。临床前数据表明，免疫治疗和化疗间可能存在协同作用。首先，化疗有很多方面的免疫调节效应，包括降低免疫抑制性细胞的数量和活性；诱导免疫原性死亡；增加肿瘤抗原呈递；激活和诱导树突状细胞成熟；增加效应T细胞功能。其次，化疗能够诱导肿瘤细胞PD-L1的表达。已有证据^[27]^证实，相比标准化疗，一线使用PD-1/PD-L1免疫检查点抑制剂联合化疗能提高晚期NSCLC患者的ORR和OS。

KEYNOTE021研究在未考虑PD-L1表达情况下，将Pembrolizumab与培美曲塞/卡铂化疗方案联合治疗转移性非鳞NSCLC。结果显示，较单纯化疗组，联合治疗组ORR提高26%，PFS和OS显著改善，同时还使疾病进展或死亡风险降低47%。这是目前为止免疫联合化疗随访时间最长的研究^[28-30]^。KEYNOTE189研究在其基础上进行深入探索发现，Pembrolizumab显著提高EGFR/ALK阴性非鳞NSCLC的ORR（47.6% *vs* 18.9%）、PFS（8.8个月*vs* 4.9个月）和OS（NR *vs* 11.3个月）。不论PD-L1表达如何，均有获益；PD-L1表达越高，获益越大^[31]^。值得注意的是，Pembrolizumab联合方案可轻微增加化疗的AEs发生率，尤其是肾脏损害；但是生活质量评分结果显示，在AEs发生率较高的情况下联合方案仍能获得较高的生活质量^[32, 33]^。美国FDA已批准Pembrolizumab联合化疗用于PD-L1（TPS≥50%）的转移性非鳞NSCLC的一线治疗^[34]^。而KEYNOTE407研究彰显了免疫联合化疗在晚期鳞状NSCLC的疗效。与单用化疗相比，Pembrolizumab联合卡铂/紫杉醇或白蛋白结合型紫杉醇显著延长OS及PFS（OS: HR=0.64, PFS: HR=0.56）。OS获益见于不同的PD-L1表达亚组：TPS < 1%（HR=0.6）、TPS1-49%（HR=0.57）、TPS≥50%（HR=0.64）。在PD-L1高表达（TPS≥50%）亚组，PFS从4.2个月提高至8.0个月，进展风险降低了63%；次要终点ORR和持续缓解时间也显著改善，绝大部分AEs发生率及严重程度相似^[33, 35]^。基于此项研究，Pembrolizumab联合卡铂/紫杉醇或白蛋白结合型紫杉醇成为一线治疗转移性鳞状NSCLC的新标准，且与PD-L1表达状态无关。

IMpower131及IMpower132研究将Atezolizumab联合化疗方案分别用于鳞状NSCLC、非鳞状NSCLC的一线治疗^[36]^。IMpower131研究分为A组（Atezolizumab联合卡铂/紫杉醇）、B组（Atezolizumab联合卡铂/白蛋白结合型紫杉醇）和C组（卡铂/紫杉醇）三组进行对比，结果显示在意向治疗（intent to treat, ITT）人群中，B组对照C组的PFS获益可见于所有PD-L1表达人群，在高表达人群中获益更为显著。这无疑为免疫治疗在晚期鳞状NSCLC的应用提供了更多的成功例证^[37]^。IMpower132研究则限定了EGFR/ALK阴性的Ⅳ期非鳞NSCLC患者，在ITT人群中，联合组（Atezolizumab联合卡铂或顺铂/培美曲塞）中位无进展生存期（median progression free survial, mPFS）和mOS均较化疗组有所提高（mPFS：7.6个月*vs* 5.2个月，HR=0.60；OS：18.1个月*vs* 13.6个月）。探索性分析发现，ORR、mPFS在PD-L1高表达和阴性亚组差异有显著统计学意义，而在低表达组差异并不显著，一些研究学者考虑这一现象与检测方法有关^[38]^。

免疫联合化疗不仅在疗效指标上取胜，而且安全性可控可管理。这为免疫治疗在更广泛人群中的应用提供了可能，更进一步完善了晚期NSCLC个体化精准治疗的蓝图。

### 3.3 二线治疗

既往治疗失败的晚期NSCLC患者应用化疗治疗有效率仅为8%-9%左右，生存预后不理想。随着对免疫治疗的探索，目前已有多项临床研究证实PD-1/PD-L1免疫检查点抑制剂在晚期NSCLC二线治疗中的临床价值^[39-41]^。与二线标准化疗方案多西他赛相比，免疫治疗对于鳞状NSCLC及非鳞状NSCLC都有明显的优势，并且一旦患者应用免疫治疗有效，PFS能够得到明显的延长。

Ⅲ期临床试验CheckMate017研究和CheckMate057研究将Nivolumab（3 mg/kg, *q2w*）与多西他赛（75 mg/kg, *q3w*）对比，分别用于治疗晚期鳞状及非鳞状NSCLC患者。在CheckMate017研究中，对比多西他赛，Nivolumab治疗鳞状NSCLC的客观缓解率ORR、mPFS和mOS均有所提高（ORR：20% *vs* 9%；mPFS：2.3个月*vs* 4.2个月，HR=0.62, 95%CI：0.47-0.81；mOS：9.2个月*vs* 6.0个月，HR=0.59，95%CI：0.44-0.79）^[42]^。在CheckMate057研究中，Nivolumab组ORR、mOS更优（ORR：19% *vs* 12%；mOS：12.2个月*vs* 9.4个月，HR=0.73，95%CI：0.59-0.89）^[43, 44]^。尽管mPFS无明显差异（2.3个月*vs* 4.2个月），1年PFS率却显著提高（19% *vs* 8%）^[45]^。正是基于这两项研究，美国FDA批准Nivolumab用于以铂类为基础化疗方案的进展晚期（转移性）鳞状及非鳞状NSCLC的治疗^[43]^。CheckMate078研究是第一个在东亚人群（主要是中国）显示出PD-1/PDL1抑制剂二线治疗晚期NSCLC具有显著OS延长的多中心、随机Ⅲ期研究，入组标准限定EGFR和ALK阴性患者，将组织病理学亚型（鳞状及非鳞状NSCLC）及PD-L1表达状况（TPS≥1% *vs* < 1%或不可评估）作为分层因素。数据显示，鳞状NSCLC患者更能从Nivolumab二线治疗中获益（ORR：16.6% *vs* 4.2%，mOS：12.0个月*vs* 9.6个月，HR=0.68，95%CI：0.52-0.90）；PD-L1低表达或不可评估的患者获益不显著^[46]^。总体而言，CheckMate078与CheckMate017/057研究结果相一致，首次证实了Nivolumab在亚洲人群中良好的疗效和安全性。2018年6月中国药品监督管理批准Nivolumab应用于EGFR和ALK阴性的晚期或转移性NSCLC二线治疗。

KEYNOTE001研究和KEYNOTE010研究则将PD-L1阳性（TPS≥1%）的晚期NSCLC患者纳入目标人群进行探索。Ib期KEYNOTE001研究应用Pembrolizumab（2 mg/kg, *q3w*或10 mg/kg, *q2w*）单药治疗取得长期生存获益，且未观察到治疗引起累积免疫相关性毒性或迟发性3级-5级毒性^[47-49]^。Ⅱ期/Ⅲ期KEYNOTE010研究将既往接受过治疗的局部晚期或转移性NSCLC患者按1:1:1随机分组，分别予以Pembrolizumab（2 mg/kg）、Pembrolizumab（10 mg/kg）或多西他赛（75 mg/kg）治疗。结果显示，应用Pembrolizumab治疗PFS更优（14.9个月*vs* 17.3个月*vs* 8.2个月），并确定了PD-L1阳性（TPS≥50%）者为最佳获益人群^[50-53]^。2014年，Pembrolizumab成功获批应用于PD-L1阳性（TPS≥50%）晚期NSCLC的二线治疗。

抗PD-L1抗体Atezolizumab相对多西他赛在ORR、OS和PFS上也表现出一定的优势。与抗PD-1抗体的作用机制不同，Atezolizumab除了阻断PD-L1和PD-1的相互作用外，还可以激活被抑制的免疫细胞、清除肿瘤细胞。同时能抑制PD-L1和B7-1的相互作用，增强抗肿瘤免疫作用^[54]^。Ⅱ期BIRCH研究分别纳入142例初治和525例经治晚期NSCLC患者。其中，在271例至少接受过一次含铂方案治疗的患者中，应用Atezolizumab治疗的ORR、6个月生存率和6个月PFS率分别为17%、76%和29%，而且PD-L1表达越高疗效越好^[26]^。POPLAR研究分别予以既往治疗失败的NSCLC患者Atezolizumab（1, 200 mg/kg）或多西他赛（75 mg/m2）治疗，对比发现Atezolizumab以2.9个月的mOS领先多西他赛（12.6个月*vs* 9.2个月，HR=0.73，95%CI：0.53-0.99）^[55, 56]^。Ⅲ期OAK临床试验进一步证实了POPLAR研究。无论PD-L1表达状态和组织学类型如何，Atezolizumab较多西他赛均有明显生存获益（OS：13.8个月*vs* 9.6个月）^[56]^；进一步亚组分析发现，PD-L1高表达者获益最佳。在安全性方面，AEs的发生率较低^[57-59]^。Atezolizumab于2016年成为第一个获批用于接受含铂化疗治疗期间或治疗后病情进展以及接受靶向治疗失败的转移性NSCLC的抗PD-L1单抗。

JAVELIN Lung 200研究将Avelumab与多西他赛进行了对比。初期分析发现，在PD-L1阳性（TPS≥1%）经治晚期NSCLC患者中没有达到主要研究终点，Avelumab对比多西他赛并没有OS的改善（11.4个月*vs* 10.3个月，HR=0.90）。考虑后续免疫检查点抑制剂的高度使用可能影响了研究中多西他赛组的OS，使其比预期的更长（8.1个月-9.6个月）。探索性分析发现，Avelumab在较高的PD-L1表达（TPS≥50%和≥80%）的患者中，表现出更强的临床活性（HR=0.6和HR=0.59）。此外，在鳞状NSCLC人群中OS有更长的趋势^[60]^。

总之，多种PD-1/PD-L1免疫检测点抑制剂已成为二线治疗NSCLC的新标准，为晚期肺癌患者提供了更多的治疗选择。遗憾的是，尽管免疫检测点抑制剂的出现使得总体生存状况得到一定程度改善，但是有效率仍只有20%左右，明显低于一线治疗的效果。如何提高二线治疗的疗效有待进一步研究。

### 3.4 同步放化疗后巩固治疗

对于病灶不可切除的Ⅲ期NSCLC，标准治疗方案是铂类为基础的放化疗，然而PFS约为8个月，5年生存率仅有15%-30%，探索更为有效的治疗方案势在必行。

PACIFIC是第一项评估免疫检查点抑制剂用于局部晚期不可手术切除的NSCLC疗效的研究。无论PD-L1表达状态如何，将入组患者按2:1随机分组，分别予以Durvalumab和安慰剂治疗，旨在探讨局部晚期NSCLC根治性同步放化疗后巩固Durvalumab的疗效和安全性。结果证实，相较安慰剂，Durvalumab治疗的主要研究终点PFS和mOS均有显著统计学意义及临床意义的延长（PFS：17.2个月*vs* 5.6个月，HR=0.51；OS：NR *vs* 28.7个月，HR=0.68）^[61]^；次要终点ORR及至死亡或远处转移时间也有所改善，且两组安全性相似^[16, 62-65]^。目前，Durvalumab已被推荐作为局部晚期NSCLC同步放化疗后巩固治疗（2A类证据）。

## 4 PD-1/PD-L1免疫检查点抑制剂在SLCL的探索

小细胞肺癌（small cell lung cancer, SCLC）是肺癌中恶性程度最高的病理类型，大多数患者诊断时即为广泛期SCLC（extensive stage-small cell lung cancer, ES-SCLC）。20多年来，ES-SCLC的治疗进展甚微，一线标准治疗方案仍为铂类（卡铂或顺铂）联合依托泊苷，尽管有效率约50%-70%，多数患者容易出现复发和耐药，mOS仅为9个月-11个月；二线治疗仅有拓扑替康获批，治疗手段相对匮乏。面对SCLC患者总体生存预后不理想的现状，各项研究陆续展开以尝试应用免疫治疗突破SCLC治疗困境^[66]^。

CheckMate032研究评估Nivolumab单药或联合Ipilimumab治疗经治复发性局限期或ES-SCLC获得理想的生存获益^[67]^。据此，Nivolumab获批可单药用于复发性SCLC三线治疗^[68, 69]^，同时Nivolumab也成为目前唯一在NSCLC和SCLC中均得到推荐的免疫药物。然而，在接受含铂化疗方案后进展的复发性SCLC患者中展开的CheckMate331研究未达到主要终点，与二线拓扑替康或氨柔比星化疗方案相比，Nivolumab未能显著延长OS^[70]^。考虑纳入人群中PD-L1高表达患者不足可能是造成试验失败的一个重要原因。

KEYNOTE028研究将Pembrolizumab单药用于PD-L1（TPS≥1%）的二线SCLC患者显示出良好的抗肿瘤潜力，其ORR达33.3%，1年PFS率为2.8%，1年生存率为37.7%，优于既往二线化疗方案^[71-73]^。KEYNOTE158研究则未考虑PD-L1表达状况进行评估Pembrolizumab治疗多种晚期实体瘤。在SCLC人群中，结果显示总体ORR为18.7%，PFS为2.0个月，OS为9.1个月；亚组分析显示PD-L1阳性患者有更好的生存获益，也预示着PD-L1可作为Pembrolizumab治疗SCLC的疗效生物标志物^[74]^。正在进行中的Pembrolizumab联合化疗一线治疗ES-SCLC的KEYNOTE-604研究结果值得期待。

IMpower133研究评估了Atezolizumab联合卡铂/依托泊苷一线治疗ES-SCLC的疗效和安全性，是首个显示OS较当前一线标准方案获得显著临床意义改善的研究（OS:12.3个月*vs* 10.3个月，HR=0.70）^[75]^。此外，中位PFS、中位持续缓解时间都有延长且不良反应可控。这些数据彰显了免疫治疗在ES-SCLC领域的重大突破，Atezolizuma联合卡铂/依托泊苷成为ES-SCLC一线治疗新标准。

总体而言，PD-1/PD-L1免疫检查点抑制剂治疗SCLC前景良好，但是免疫治疗在SCLC领域的探索之路依然任重道远，尚需进一步筛选优势人群及优化治疗方案评估其治疗效果。

## 5 PD-1/PD-L1免疫检查点抑制剂的机遇与挑战

### 5.1 免疫联合化疗

已有不少临床研究获得免疫联合取胜的结果^[76]^。就免疫联合化疗而言，KEYNOTE021与KEYNOTE189研究显示Pembrolizumab联合培美曲塞/铂类较单用化疗显著延长非鳞NSCLC的PFS、OS，KEYNOTE-407研究显示Pembrolizumab联合卡铂/紫杉醇或白蛋白结合型紫杉醇较单用化疗显著改善鳞状NSCLC的PFS、OS。Pembrolizumab作为一线药物联合化疗治疗EGFR/ALK阴性的晚期NSCLC人群可全面降低死亡风险，且不良反应未见增加。进一步探索发现，KEYNOTE189研究中，Pembrolizumab联合培美曲塞/卡铂与培美曲塞/顺铂两种方案相比，疗效均有增加，卡铂和顺铂组无显著差异。KEYNOTE407研究中将白蛋白紫杉醇/卡铂与紫杉醇/卡铂方案相比，结果显示，两组的ORR均明显提高，OS显著延长，之间亦未见明显差异。

免疫联合治疗蓬勃发展，然而，目前为止，相关疗效对比研究主要集中在同类药物之间的比较，而对于联合不同化疗方案之间是否具有疗效差异，目前尚无研究结果证实。免疫联合治疗在化疗方案选择上，需要考虑以下方面：（1）不同化疗方案本身具有疗效差异。临床工作中，不同病理类型肺癌首选化疗方案并不一致，例如，鳞状NSCLC常采用吉西他滨/铂类，而对于非鳞NSCLC培美曲塞/卡铂方案疗效更佳^[77]^。联用免疫治疗之后，原有首选方案是否仍是最佳选择，值得进一步探究。（2）化疗药物对免疫功能的影响。不同化疗药物对免疫系统的影响也略有不同，例如，培美曲塞能够选择性激活NK细胞产生IFN-γ^[78]^，紫衫烷类能够降低Treg活性^[79]^。免疫治疗的加入放大了免疫系统在肿瘤治疗过程中的作用，难免牵一发而动全身，因此在联合方案选择上，化疗药物对免疫功能的影响同样需要考虑在内。（3）化疗药物的骨髓抑制。多数化疗药物对患者骨髓功能具有抑制作用，部分药物更是能够引起严重骨髓抑制。严重骨髓抑制必然会对免疫治疗疗效产生负面影响，因此在方案选择上需要选择骨髓抑制作用较轻的药物。（4）药物配伍作用。部分化疗药物使用过程中需要与其他药物配伍使用，对于易引发过敏反应的药物，应用糖皮质激素是常见预处理方式。这些包括激素在内的配伍药物同样能够对机体免疫功能产生影响，因此是在化疗方案选择中另一不可忽视的因素。除此之外，免疫治疗解除了免疫抑制，同时化疗杀伤肿瘤细胞释放大量肿瘤抗原，引发特异性免疫反应。然而一旦免疫功能过度激活，难免会引起蝴蝶效应，乃至引发免疫风暴，需要临床医生警惕此类事件的发生。

### 5.2 免疫联合其他治疗

由于联合治疗的巨大潜力，越来越多的相关临床研究结果带来惊喜。例如，IMpower150将免疫联合化疗及抗血管生成治疗用于晚期鳞状NSCLC的一线治疗。结果显示在卡铂/紫杉醇联合贝伐珠单抗中加入Atezoliumab显著延长了PFS，其获益可见于所有人群（包括*EGFR*/*ALK*突变阳性、Teff低表达、PD-L1阴性和肝转移患者），且安全可耐受^[80]^。然而，也有一些免疫联合靶向的研究出现了疗效不增、毒性加大的表现。例如，Ib期WP29158研究应用Atezolizumab联合Erlotinib一线治疗*EGFR*突变NSCLC，安全性分析显示50%的患者出现严重AEs，与治疗相关的3级/4级AEs占39%。TATTON和CAURAL研究已因间质性肺毒性发生率过高终止研究^[81]^。

因此，联合治疗的方案选择、最佳联合治疗策略、联合治疗的应用时机和最佳剂量尚需深入研究。

### 5.3 生物标志物探索

探索行之有效的疗效预测手段筛选潜在获益人群，是免疫治疗中的关键步骤。然而，现有的生物标志物虽价值颇丰但不完美^[82]^。

目前，PD-L1检测已经写入NCCN-NSCLC指南，其表达对疗效预测具有一定指导意义^[83]^，但是不完全准确^[84, 85]^。PD-L1表达具有空间异质性及时间异质性，面临的诸多挑战主要体现在：（1）PD-L1表达与疗效的相关性：既往研究结果发现，部分人群PD-L1表达阴性，仍可以从免疫治疗中获益，而部分PD-L1表达阳性人群不能获益或获益不明显，如CheckMate026试验中PD-L1（TPS≥50%）人群的PFS和OS仍无获益。因此，尚需考虑是否所有患者均需要检测PD-L1表达？对于PD-L1表达阴性人群，是否需要使用免疫抑制剂？（2）有效性的界定：主要包括截断值的判定（即PD-L1的表达强度，多少百分比为“阳性”），从而进一步探索最佳截断值。还包括表达PD-L1的细胞类型、部位、分布等。（3）检测标准的严格性：PD-L1表达情况何时检测，何种手段检测，尚无统一标准。现有的检测平台和试剂各不相同，Nivolumab、Pembrolizumab和Avelumab分别采用Dako28-8、22C3和73-10，Atezolizumab和Durvalumab分别采用VENTANA SP142和SP263。为探索在抗PD-1/PD-L1治疗检测方法的可靠性，检测平台和抗体的一致性研究如蓝印计划（The Blueprint Project）、德国一致性研究（German Round Robin）、AZ比较研究（AZ Comparative Study）相继展开。

此外，TMB也成为最具潜力的免疫治疗生物标志物之一^[86]^。Checkmate026研究探索性分析发现，高TMB者应用Nivolumab治疗PFS有临床获益；PD-L1表达（TPS≥50%）且高TMB患者PFS获益最为明显。KEYNOTE001研究结果提示，Pembrolizumab疗效与TMB呈正相关，79%的高TMB患者具有持久临床获益。POPLAR/OAK研究显示Atezolizumab疗效与外周血TMB成正相关。然而，TMB作为免疫治疗生物标志物也具有一定局限性。如缺乏前瞻性研究证据；费用昂贵，技术复杂，尚处于研究阶段；组织TMB与外周血TMB是否具有一致性；截断值如何设定等。

鉴于免疫反应的调控机制非常复杂，仅凭单一的生物标志物似乎并不够。因此，进行疗效预测因子的多因素分析、关键生物标志物的联合可能在未来的应用中更加可行。

### 5.4 不良反应的管理

免疫相关AEs（immune-related adverse events, irAEs）定义为通过靶向CTLA-4和PD-1及其配体PD-L1的免疫检查点阻断剂使用，产生了一系列因免疫细胞（尤其是T细胞）组织浸润而导致的独特的毒性反应。

免疫治疗呈现出来的某些AEs或许类似于其它治疗模式，但是相似的AEs可能有着不同机制，例如腹泻和肠炎、皮疹和瘙痒等。irAEs的发生与炎症反应有关，特别是CD8^+^T细胞激活介导的炎症反应，其他类型的炎症细胞（如Th17），也可能参与其中。既往研究指出，受损皮肤和内脏的免疫组化显示CD4^+^和CD8^+^T细胞浸润，并且高度激活的效应细胞与AEs发生有一定的相关性^[5, 87-89]^。irAEs分为三类：（1）器官特异性AEs：结肠炎、肝炎、肺炎、甲低等；（2）一般性AEs：疲乏、腹泻、皮疹等；（3）其他可能与系统性炎症相关的AEs。大多数irAEs轻到中度，但也有严重或危及生命的现象发生，以神经系统和心脏引起的致死率最高^[90]^。与传统化疗相比，irAEs出现延迟、持续时间更长，且毒性谱不同，以肺炎、甲减、关节痛等更常见^[5, 56, 90-92]^。研究发现，PD-1/PD-L1抑制剂相对化疗，AEs整体发生率较低，与治疗相关的3级/4级AEs占7%-13%，安全性较高^[44, 53, 56]^。而联合治疗后AEs发生率有所升高，但可耐受^[32, 33]^。目前免疫治疗的毒性管理指南和共识已陆续发布，大多数irAEs可以通过暂停给药加或不加皮质类固醇激素得以控制，且可以逆转^[93, 94]^。

总之，irAEs发病隐匿，缺乏特异性，毒性谱广，需要临床医生从预防、评估、检查、治疗、检测五大环节加强irAEs的管理，从而有效控制病情。

### 5.5 驱动基因突变人群

CheckMate057、KEYNOTE010和OAK试验亚组分析分别显示*EGFR*突变患者并未能从Nivolumab、Pembrolizumab和Atezolizumab二线治疗中获益。既往*meta*分析也发现*EGFR*突变可能是免疫治疗的负向指标^[95]^。目前，对于驱动基因突变的NSCLC能否使用免疫治疗尚有争议。对于*EGFR*敏感突变率较高的中国肺癌患者，免疫治疗是否可行？该如何使用？目前在研临床研究的研究模式是否适合中国人群？EGFR-TKI耐药后给予免疫治疗能否获益？这需要开展更多的临床研究进一步证实。

## 6 结论

免疫治疗的发展如火如荼，可喜可贺的临床获益为晚期肺癌患者带来曙光。但是，受益人群比例较低且面临着诸多挑战。目前中国免疫治疗基础研究成果较多，尚缺乏转化及临床研究的投入，还需要展开大量的多中心大型试验。结合中国肺癌人群的特点及分布，筛选出潜在优势人群，探索低毒高效的最佳治疗策略，并进一步扩大获益人群。
